# The parasite specific substitution matrices improve the annotation of apicomplexan proteins

**DOI:** 10.1186/1471-2164-13-S7-S19

**Published:** 2012-12-07

**Authors:** Jamshaid Ali, Shashi Rekha Thummala, Akash Ranjan

**Affiliations:** 1 Laboratory of Computational and Functional Genomics, Centre for DNA Fingerprinting and Diagnostics (CDFD), A Sun Centre of Excellence in Medical Bioinformatics, Tuljaiguda, Nampally, Hyderabad 500001, India

## Abstract

**Background:**

A number of apicomplexan genomes have been sequenced successfully in recent years and this would help in understanding the biology of apicomplexan parasites. The members of the phylum Apicomplexa are important protozoan parasites (*Plasmodium, Toxoplasma *and *Cryptosporidium *etc) that cause some of the deadly diseases in humans and animals. In our earlier studies, we have shown that the standard BLOSUM matrices are not suitable for compositionally biased apicomplexan proteins. So we developed a novel series (SMAT and PfFSmat60) of substitution matrices which performed better in comparison to standard BLOSUM matrices and developed ApicoAlign, a sequence search and alignment tool for apicomplexan proteins. In this study, we demonstrate the higher specificity of these matrices and make an attempt to improve the annotation of apicomplexan kinases and proteases.

**Results:**

The ROC curves proved that SMAT80 performs best for apicomplexan proteins followed by compositionally adjusted BLOSUM62 (PSI-BLAST searches), BLOSUM90 and BLOSUM62 matrices in terms of detecting true positives. The poor E-values and/or bit scores given by SMAT80 matrix for the experimentally identified coccidia-specific oocyst wall proteins against hematozoan (non-coccidian) parasites further supported the higher specificity of the same. SMAT80 uniquely detected (missed by BLOSUM) orthologs for 1374 apicomplexan hypothetical proteins against SwissProt database and predicted 70 kinases and 17 proteases. Further analysis confirmed the conservation of functional residues of kinase domain in one of the SMAT80 detected kinases. Similarly, one of the SMAT80 detected proteases was predicted to be a rhomboid protease.

**Conclusions:**

The parasite specific substitution matrices have higher specificity for apicomplexan proteins and are helpful in detecting the orthologs missed by BLOSUM matrices and thereby improve the annotation of apicomplexan proteins which are hypothetical or with unknown function.

## Background

One of the most important and challenging tasks of post-genomic era is to improve the annotation of newly sequenced genomes in general and of parasite genomes in particular. The members of the phylum Apicomplexa are important protozoan parasites that cause some of the deadly diseases in humans and animals [1,2]. They include parasites like *Plasmodium, Toxoplasma, Eimeria, Neospora, Cryptosporidium, Babesia *and *Theileria*. Apicomplexan genomics started with the completion of *Plasmodium falciparum *genome sequence [3] and no homology was detected for approximately 60% of its genes [3]. Later, a number of apicomplexan parasite genomes were sequenced successfully followed by genome annotation projects which would help in understanding the biology of these parasites [4-8]. The amino acid substitution and composition in *P. falciparum *proteins were unusual and standard matrices (BLOSUM & PAM) did not detect orthologs and/or gave poor alignment for many *P. falciparum *proteins [9-11]. In order to address this issue we developed an alternate option *i.e*. a novel series of substitution matrices (SMAT and PfFSmat60) and demonstrated their superior performance over the standard matrices (BLOSUM and PAM) for *P. falciparum *proteins in particular [9] and for apicomplexan proteins in general [10]. We further demonstrated that the amino acid compositions of proteins of nine apicomplexan parasites (*Toxoplasma gondii, Neospora caninum, Theileria parva, Cryptosporidium parvum, P. berghei, P. chabaudi, P. knowlesi, P. vivax *and *P. yoelii yoelii*) were similar to that of *P. falciparum *and because of this unusual amino acid composition of apicomplexan proteins these matrices (originally developed for *P. falciparum*) performed better even for other apicomplexan proteins (when compared to standard matrices BLOSUM & PAM) [10]. Moreover to provide access to this novel series of matrices to researchers working on apicomplexan parasites, a web server ApicoAlign (http://www.cdfd.org.in/apicoalign/) was developed to detect orthologs and align apicomplexan proteins [10]. In the present study, we assess the performance of these matrices with that of compositionally adjusted matrices (sensitive PSI-BLAST searches) in terms of detection of the true and false positives, an important aspect missing in our earlier studies [9,10]. Many protein families like kinases are under-represented in apicomplexan parasites probably because standard matrices (BLOSUM & PAM) could not detect them during genome annotation. SMAT80 uniquely detected (*i.e*. missed by BLOSUM matrices) completely or partially annotated ortholog proteins for 1374 apicomplexan hypothetical proteins against SwissProt database.

## Results and discussion

### SMAT80 detected more true positives

**Figure 1:**
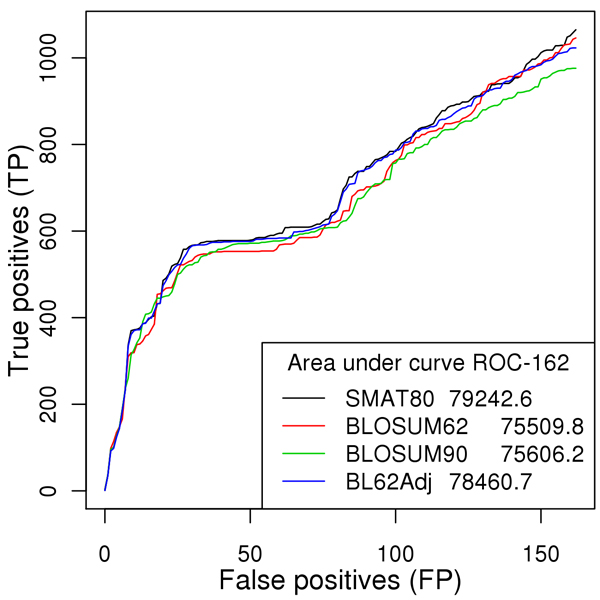
**ROC162 plot of PbGO vs. PyGO BLAST searches**. ROC curves, at a cut-off of 162 false positives (FP), were calculated for BLOSUM62, BLOSUM90, SMAT80 and compositionally adjusted BLOSUM62 matrices. In the legend, AUC162 values, corresponding to the area under ROC curves, are reported.

In order to assess the performance of different matrices in terms of true and false positives, we used the method adopted by Brick and co-workers [11] (described in Methods). In general, SMAT80 performed best (see AUC162 values in parentheses and black line in Figure 1), followed by the compositionally-adjusted BLOSUM62 (blue line in Figure 1), BLOSUM90 (green line in Figure 1) and BLOSUM62 (red line in Figure 1). In more detail, all ROC162 curves in Figure 1 are very alike in the initial regions and this is expected as all the examined matrices perform similarly when aligning highly similar proteins. In fact, the first part of the curves corresponds to hits with high bit scores and low E-values. However, in the latter region, ROC162 curves diverge from each other. The number of false positive hits increases steeply for BL62adj (compositionally adjusted BLOSUM62 matrix) while the other matrices show a less dramatic increase and thus show better performance, particularly apicomplexan specific matrix SMAT80. The overall positive predictive values (PPV = TP/TP+FP) are 40.28%, 31.87%, 31.47% and 27.26% for SMAT80, compositionally adjusted BLOSUM62Adj, BLOSUM90 and BLOSUM62 matrices respectively. Therefore SMAT80 performs best for apicomplexan proteins followed by BLOSUM62Adj, BLOSUM90 and BLOSUM62 matrices.

### SMAT80 gave poor scores for non-specific hits and better scores for specific hits

In our earlier studies [9,10] and this study, we have demonstrated that SMAT80 gave better E-values and better bit scores for most of the apicomplexan proteins. Next, we were interested to know whether parasite specific matrices would give poor E-values and/or bit scores for the proteins for which orthologs must not exist (in biological context) in a particular family/class of Apicomplexa. The phylum Apicomplexa is divided into two major classes e.g. aconoidasida (*P. berghei, P. chabaudi, P. falciparum, P. knowlesi, P. vivax, P. yoelii yoelii, B. bovis, T. annulata *and *T. parva*) and coccidia (*C. hominis, C. muris, C. parvum, E. tenella, N. caninum *and *T. gondii*). The coccidians generate a thick walled oocyst stage that is excreted with faeces while in hematozoans (members of class aconoidasida) oocysts are not excreted but transmitted by mosquito or tick [1,2,12]*i.e., *the transmissible cyst stage of coccidians is environmentally durable outside the host [13]. Because of this feature there are some proteins like oocysts wall proteins which are found exclusively in class coccidia but not in hematozoans [1,2,12,13]. Sanderson and coworkers [14] identified 52 proteins (probably involved in surface interactions) isolated from purified oocysts of *C. parvum *using 2-D gel electrophoresis and MudPIT analysis. This dataset of 52 proteins had six oocysts wall proteins (COWP1, COWP2, COWP3, COWP4, COWP6 and COWP8). We analyzed the E-values and bit scores given by SMAT80 and BLOSUM62 matrices for these six oocyst wall proteins against other apicomplexan species. We observed that the average bit scores given by SMAT80 were poor compared to BLOSUM62 for these oocyst wall proteins against hematozoan parasites but better against cryptosporidia and *Toxoplasma gondii*; and in fact these were quite high against cryptosporidia (Table 1). Since these proteins were from purified oocysts of *C. parvum*, we expected to find the true orthologs for them in other coccidian/cryptosporidia but not necessarily in hematozoans and that is why SMAT80 correctly gave poor average bit scores against hematozoans. One of the oocyst wall proteins COWP2 (cgd7_1800) gave BLAST hits against 14 apicomplexan species using SMAT80 and BLOSUM62 matrices but the hits obtained using SMAT80 matrices had poor E-values compared to those obtained using BLOSUM matrices. For example, BLOSUM62 and SMAT80 detected a common best hit for COWP2 (cgd7_1800) in *P. falciparum *and that was PFC1045c. However, BLOSUM62 and SMAT80 gave E-values of 3e-06 and 0.033 respectively for the same pair (Additional File 1). Treeck and coworkers [15] detected the same protein PFC1045c in parasite blood stages using mass spectrometry suggesting that it was not an oocyst protein. Many a times against hematozoans (*B. bovis, T. annulata, T. parva, P. berghei, P. chabaudi, P. knowlesi *and *P. vivax*), SMAT80 and BLOSUM62 matrices detected two different hits for COWP2. However, against other coccidians both matrices BLOSUM62 and SMAT80 detected a single best hit (*i.e*. true ortholog) for COWP2 and those subject hits were Chro.70210 (*C. hominis*), CMU_033840 (*C. muris*), ETH_00012470 (*E. tenella*), NCLIV_011890 (*N. caninum*) and TGME49_010950 (*T. gondii*) at E-value threshold 1e-10 (Additional File 1). It was expected because all the coccidians should have oocyst wall proteins but not aconoidasida. Therefore in this case SMAT80 correctly gave poor E-values compared to BLOSUM62 matrix. SMAT80 gave poor E-values (compared to BLOSUM62) for other oocyst wall proteins too against members of class aconoidasida and these oocyst wall proteins were COWP1 (cgd6_2090), COWP3 (cgd4_670), COWP4 (cgd8_3350), COWP6 (cgd4_3090) and COWP8 (cgd6_200) (data not shown). These examples prove that indeed SMAT80 matrix gives less false positives (or more true positives) as predicted by ROC curves (Figure 1) thereby it has better specificity than that of BLOSUM matrices.

**Table 1:** Average bit scores for *C. parvum *proteins of purified oocysts.

Subject organism	Average bit score with SMAT80	Average bit score with BLOSUM62
*Babesia bovis*	37.23	39.61
*Theileria annulata*	38.43	42.15
*Theileria parva*	39.17	41.71
*Plasmodium berghei*	44.12	49.15
*Plasmodium chabaudi*	44.72	49.4
*Plasmodium falciparum*	45.63	50.53
*Plasmodium knowlesi*	44.41	46.49
*Plasmodium vivax*	43.76	46.57
*Plasmodium yoelii yoelii*	44.14	48.55
*Cryptosporidium hominis*	1836.58	1395.09
*Cryptosporidium muris*	765.025	703.163
*Eimeria tenella*	80.6962	99.10
*Neospora caninum*	93.652	102.58
*Toxoplasma gondii*	113.69	105.66

### Genome-wise BLAST searches

**Figure 2:**
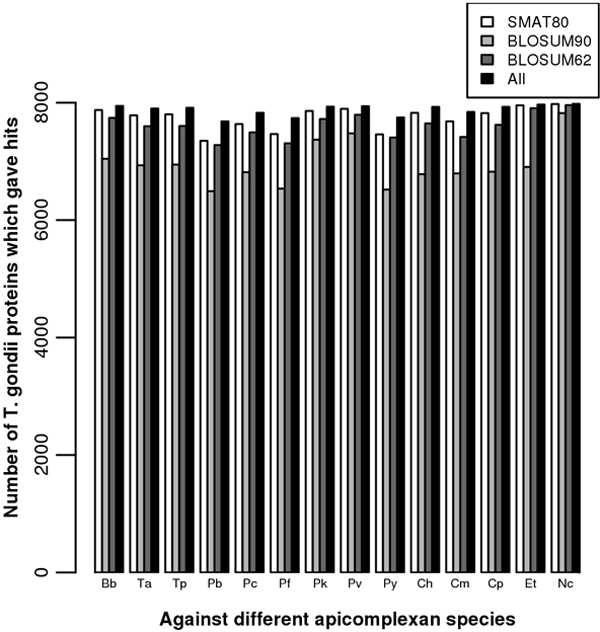
**Number of orthologs detected using different matrices**. The histogram represents the number of *Toxoplasma gondii *proteins which give orthologs using a particular matrix (given in legend) against subject apicomplexan genomes given on X-axis (the abbreviations on X-axis: Bb, Ta, Tp, Pb, Pc, Pf, Pk, Pv, Py, Ch, Cm, Cp, Et and Nc are for *Babesia bovis, Theileria annulata, Theileria parva, Plasmodium berghei, Plasmodium chabaudi, Plasmodium falciparum, Plasmodium knowlesi, Plasmodium vivax, Plasmodium yoelii yoelii, Cryptosporidium hominis, Cryptosporidium muris, Cryptosporidium parvum, Eimeria tenella *and *Neospora caninum *respectively).

The reciprocal genome-wise BLAST searches were carried out for all the proteins of 15 apicomplexan species using SMAT80, BLOSUM90 and BLOSUM62 matrices against 1215 bacterial species. The numbers of apicomplexan proteins giving hits using SMAT80 matrix against bacteria were more compared to that given by using BLOSUM series of matrices (Additional File 2). However, a large number of apicomplexan proteins did not give any BLAST hit with significant E-value against bacteria irrespective of the matrix used. Next, the genome-wise BLAST searches were carried out for 15 apicomplexan species against one another using SMAT80, BLOSUM90 and BLOSUM62 matrices. We estimated the number of proteins which gave BLAST hits against the subject genome at different E-value thresholds (0, 1e-100, 1e-50, 1e-20, 1e-10, 1e-05, 1e-01 and no cut-off) (Additional File 3). For example SMAT80, BLOSUM90 and BLOSUM62 detected orthologs for 7785, 6934 and 7599 proteins of *Toxoplasma gondii *against *Theileria annulata *respectively without any E-value cut-off and these numbers were 435, 369 and 337 respectively with a stringent E-value cut-off (1e-100) (Figure 2 & Additional File 3). In most cases SMAT80 detected more number of orthologs compared to BLOSUM90 and BLOSUM62 matrices. Therefore we expect that the BLAST searches using SMAT80 matrix would improve the annotation of apicomplexan proteins particularly those for which BLOSUM matrices do not detect any orthologs.

### Database searches

**Figure 3:**
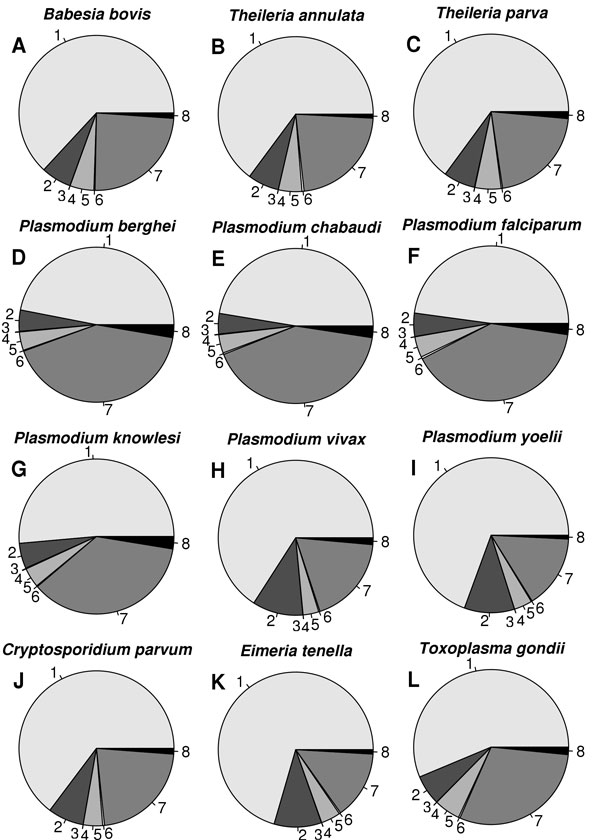
**Comparison of performance of SMAT80 with that of BLOSUM62**. We carried out BLAST searches for all the proteins of 15 apicomplexan parasites using SMAT80 and BLOSUM62 matrices against SwissProt database. An identical hit (best non-self) was assigned to one of the eight categories (1) better or similar E-values, better or similar scores and better or similar % identity with SMAT80 compared to BLOSUM62, (2) better or similar E-values, better or similar scores and poor % identity, (3) better or similar E-values, poor scores and better or similar % identity, (4) better or similar E-values, poor scores and poor % identity, (5) poor E-values, better or similar scores and better or similar % identity, (6) poor E-values, better or similar scores and poor % identity, (7) poor E-values, poor scores and better or similar % identity and (8) poor E-values, poor scores and poor % identity. As evident in the figure, most apicomplexan proteins fall in 1 & 7 categories *i.e*. SMAT80 performs better.

BLAST searches were carried out for all the proteins of 15 apicomplexan species using SMAT80, BLOSUM90 and BLOSUM62 matrices against SwissProt database. The identical hits (best non-self hits) detected by SMAT80 and BLOSUM90 matrices were compared in terms of E-values, bit scores and percent identities. These hits were classified into eight categories (described in Methods) and for each category, the percentage was calculated for all the 15 apicomplexan parasites and is shown as pie charts in Figure 3. In *Toxoplasma gondii*, SMAT80 matrix (when compared to the most commonly used matrix BLOSUM62) gave better or similar E-values, better or similar scores and better or similar % identities for 3878 proteins while it gave poor E-values, poor scores and poor % identities for only 108 proteins. Similarly, SMAT80 performed better for other apicomplexan species also (Figure 3). We compared the performance of SMAT80 matrix with that of BLOSUM90 also and SMAT80 performed better (Additional File 4).

**Figure 4:**
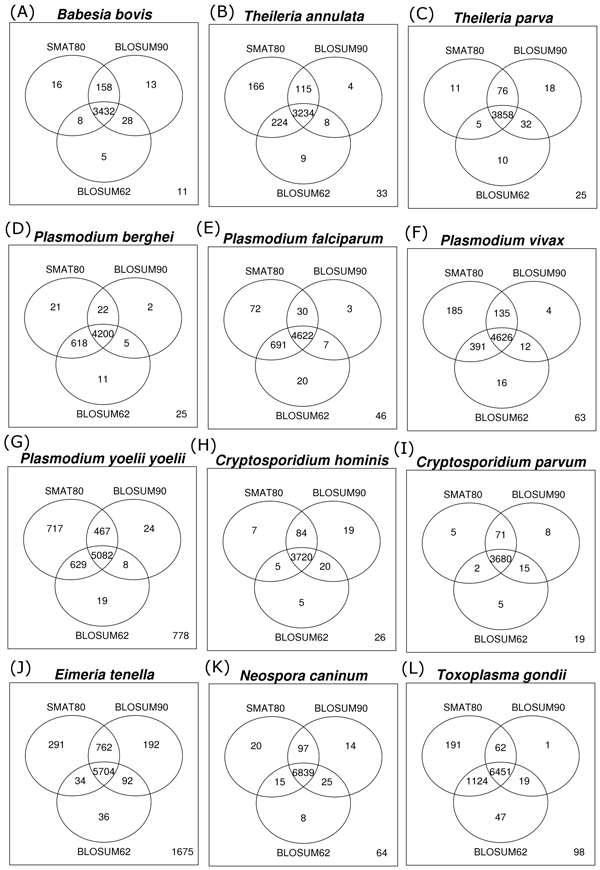
**Venn diagrams for orthologs detected against SwissProt database using different matrices**. BLAST searches were carried out against SwissProt for all the proteins of 15 apicomplexan species using SMAT80, BLOSUM90 and BLOSUM62 matrices at default E-value cut-off. The number given at bottom right corner of each Venn diagram indicates the number of proteins for which all three matrices could not detect ortholog against SwissProt database.

BLAST searches against SwissProt database were carried out for all the proteins (irrespective of their annotation status) of 15 apicomplexan parasites. Next, we estimated for how many proteins of the 15 apicomplexan species (against SwissProt database) all the three matrices were able to identify orthologs, any two matrices were able to identify orthologs and for how many proteins, only one matrix was able to identify orthologs. As we can observe in the various Venn diagrams (Figure 4), all the three matrices identified orthologs for majority of the apicomplexan proteins however if we look at the numbers of orthologs uniquely identified by a single matrix, SMAT80 performs better in comparison to BLOSUM90 & BLOSUM62 matrices. For example in *E. tenella*, SMAT80, BLOSUM90 and BLOSUM62 uniquely identified orthologs for 291, 192 and 36 proteins respectively. SMAT80 detected orthologs for more number of proteins of *T. annulata, P. berghei, P. chabaudi, P. falciparum, P. knowlesi, P. vivax, P. yoelii yoelii, E. tenella, N. caninum *and *T. gondii*. However in the case of *Babesia bovis, Theileria parva, Cryptosporidium hominis, Cryptosporidium muris *and *Cryptosporidium parvum *BLOSUM90 performs marginally better than SMAT80 (Figure 4). The data for *Plasmodium chabaudi, Plasmodium knowlesi *and *Cryptosporidium muris *are not shown. Therefore, the comparisons of SMAT80 with BLOSUM90 and BLOSUM62 clearly show that SMAT80 was able to identify orthologs for more number of apicomplexan proteins against SwissProt database and with better E-values and better bit scores.

### Apicomplexan protein hits uniquely detected by SMAT80

BLOSUM62 (default option in BLAST) is the most commonly used matrix for detecting orthologs. However we have shown that the choice of matrices can also significantly improve the ortholog detection in our previous [9,10] and the present studies. SMAT80 uniquely detected orthologs for 16, 166, 11, 21, 32, 72, 31, 185, 717, 7, 3, 5, 291, 20 and 191 proteins of *Babesia bovis, Theileria annulata, Theileria parva, P. berghei, P. chabaudi, P. falciparum, P. knowlesi, P. vivax, P. yoelii yoelii, Cryptosporidium hominis, Cryptosporidium muris, Cryptosporidium parvum, Eimeria tenella, Neospora caninum *and *Toxoplasma gondii *respectively (Figure 4). For these 1768 apicomplexan proteins, BLOSUM62 and BLOSUM90 could not identify any ortholog against SwissProt database and 1374 (out of 1768) are labeled as hypothetical proteins in EuPathDB version 2.14, the list of these proteins and their subject hits along with % identity, E-value and score are provided in Additional File 5. The annotation of SMAT80 hits (BLAST hits detected using SMAT80 matrix) for these apicomplexan proteins include 70 kinases, 14 phosphatases, 3 heat shock proteins, 17 proteases and several other proteins.

### SMAT80 detected more apicomplexan kinases

The eukaryotic protein kinases (ePKs) belong to a very extensive family of proteins which play crucial roles in most of the cellular pathways [16,17]; therefore apicomplexan kinases represent potential drug targets [18]. Ward and coworkers carried out exhaustive analysis of *P. falciparum *kinome and surprisingly found only 65 typical ePKs as *Saccharomyces cerevisiae *genome is half the size of *P. falciparum *genome but encodes approximately twice number of ePKs [18]. We speculate perhaps the standard BLOSUM matrices were not able to detect orthologs for many malarial protein kinases because of unusual amino acid composition [9,10] of apicomplexan proteins. And in fact, a novel family (FIKK) of protein kinases was reported [18] and Schneider and coworkers [19] detected many other kinases of the same family and they [18,19] considered it as Apicomplexan-specific protein kinase family.

**Figure 5:**
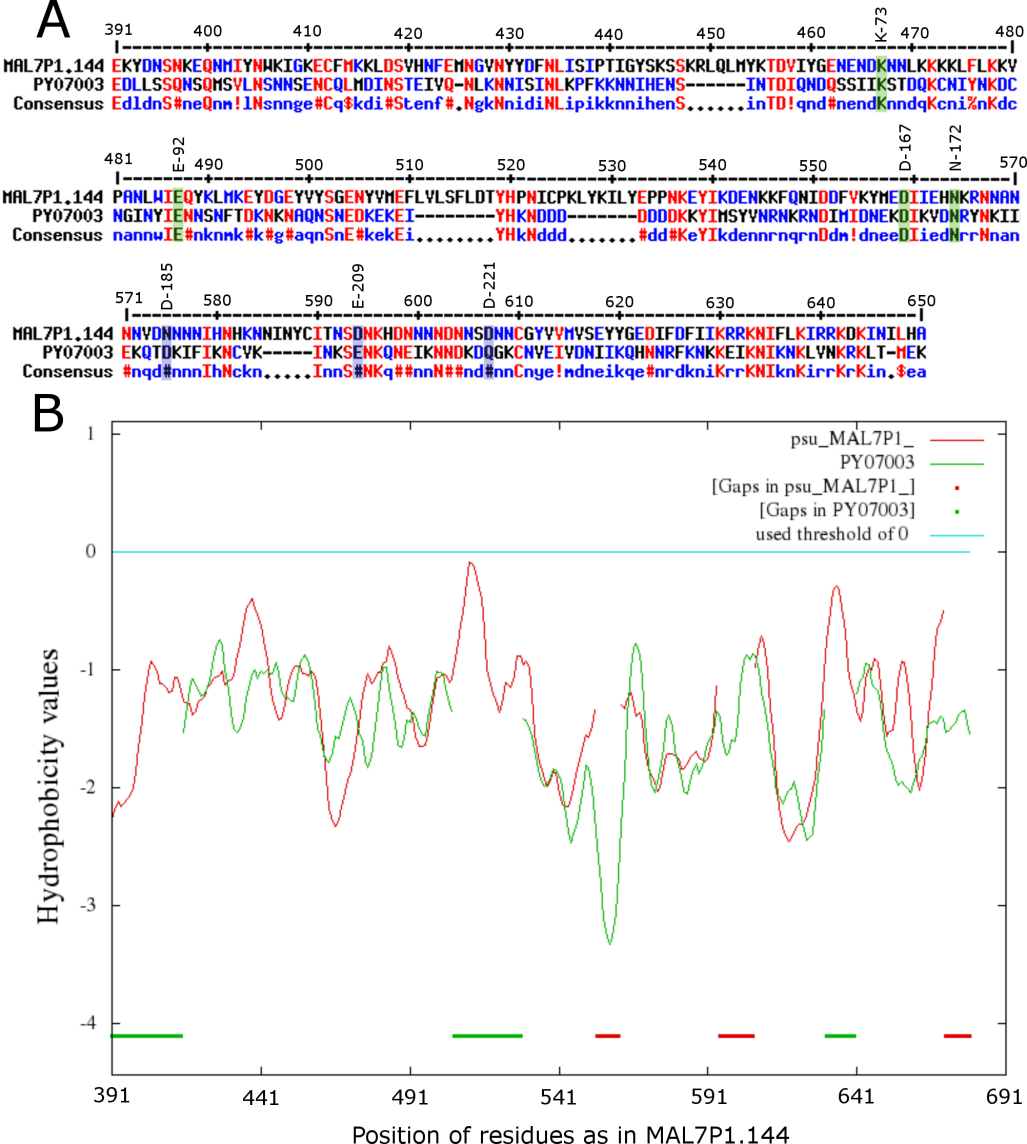
**The conserved key residues of protein kinase catalytic domain in PY07003**. (A) Multiple sequence alignment shows conservation of key residues of protein kinase catalytic domain in PY07003 (K73, E92, D167, N172, D185, E209 and D221). The kinase domain is at C-terminal in both query and subject proteins and only this part has been shown in the alignment (from 391 to 650 residues of MAL7P1.144) to highlight the conservation of important residues of kinase domain. (B) Hydrophobicity profiles of same regions of both proteins show approximately 70% matched positions.

SMAT80 detected orthologs for 1374 apicomplexan hypothetical proteins which did not give any hit against SwissProt database using BLOSUM series of matrices. The SwissProt annotation of 70 subject hits (out of 1374) is protein kinase activity (Additional File 6); that means SMAT80 predicts these 70 apicomplexan proteins (presently labeled as hypothetical proteins in EuPathDB version 2.14) as probable protein kinases. We carried out conserved domain search (in batch mode) at NCBI site for these 70 proteins but could find hits only for 8 proteins (Additional File 7) and no kinase domain was detected. However when we aligned these proteins with an experimentally known *P. falciparum *protein kinase (PF11_0220, protein kinase activity, Molecular Function GO:0004672, evidence code: Inferred by Direct Assay (IDA), source: PlasmoDB release 9.0) using PfFSmat60 matrix [9,10], the alignments were significantly lengthier (Additional File 8) suggesting these proteins were probably protein kinases. PY07003 was one such apicomplexan hypothetical protein and its subject hit (E-value 9e-11) was a serine/threonine protein kinase of *Dictyostelium discoideum *(Q55FT4). We further observed that the key residues of protein kinase catalytic domain (K73, E92, D167, N172, D185, E209 and D221) were conserved in PY07003. The lysine in subdomain II (K73) plays a role in contacting α and β phosphates of ATP, anchoring and orienting the ATP; the glutamate of subdomain III (E92) forms a salt bridge with K73; aspartate (of conserved residues D167 & N172, it is actually a signature motif HRDXXXXN of ePKs in subdomain VIB) is the catalytic residue acting as a base receptor; the aspartate in the subdomain VII (D185) binds to the Mg^2+ ^(or Mn^2+^) ion associated with the β and γ phosphates of ATP; the glutamate in subdomain VIII (E209) forms a salt bond with the arginine in subdomain XI; and the aspartate in subdomain IX (D221) is involved in structural stability of the catalytic loop of the subdomain VI through hydrogen bonding with the backbone [16-18]. In fact, all these functional residues for kinase activity were conserved in PY07003 except the Glycine triad (GxGxxG) in subdomain I and Ward and coworkers [18] too reported its absence in FIKK-family. The pairwise alignment of PY07003 with FIKK-family protein kinase of *P. falciparum *(MAL7P1.144) shows the conservation of these functional residues in Figure 5A. In addition to this, we have provided the list of apicomplexan hypothetical proteins whose subject annotations include 'kinase' after combining the BLAST hits of SMAT80, BLOSUM90 and BLOSUM62 matrices (*i.e*. union of the three matrices) (Additional File 9). We also calculated the GRAVY (grand average of hydropathy) values for these SMAT80-predicted kinases (described in Methods). Out of these 70 SMAT80-predicted protein kinases, we found that eight kinases; PY05823 (1.848), PY05872 (0.779), PY07287 (0.359), PY07161 (0.353), PY07667 (0.237), PY06969 (0.212), ETH_00018415 (0.177), PY03046 (0.174) had positive values indicating their hydrophobic nature while the remaining 62 had negative values ranging from -0.002 (TA04215) to -1.688 (ETH_00037830) indicating their hydrophilic nature. The serine/threonine protein kinase tsuA (Q55FT4) of *D. discoideum *(subject hit of SMAT80 predicted kinase PY07003) had a GRAVY value of -0.731 which was negative suggesting a hydrophilic nature, as was the case with 62 (out of 70) SMAT80-predicted kinases (Additional File 10). We would also like to mention that the serine/threonine protein kinase tsuA (Q55FT4) of *D. discoideum *is a reviewed entry in SwissProt database with a clear experimental evidence for the existence of protein. The kinase domain is at C-terminal in both MAL7P1.144 and PY07003 proteins that is why we have shown the alignment in C-terminal part (Figure 5A) and hydrophobicity profile of this alignment showed approximately 70% matched positions in terms of hydrophobicity (Figure 5B). The prediction of 70 apicomplexan probable kinases would be useful in understanding the apicomplexan kinomes as completion of the same for completely sequenced genomes is also one of the important goals of post-genomic era.

### Apicomplexan proteases missed by BLOSUM but detected by SMAT80

Several studies [20-24] have suggested that proteases are important for invasion by apicomplexan parasites. Wu and coworkers [25] revealed hidden families of proteases in malaria parasite genome and completion of apicomplexan genomes provides a basis for identifying new proteases. The SwissProt hits uniquely detected by SMAT80 for 17 apicomplexan hypothetical proteins (Additional File 11) have protease annotation *i.e*. SMAT80 predicts these hypothetical proteins as proteases. The conserved domain search in batch mode at NCBI site was carried out for these 17 proteins but could find hits only for 8 proteins. PVX_114890 (presently labeled as conserved hypothetical protein in PlasmoDB version 9.0) gave hits for rhomboid superfamily of proteases (Additional File 12) in this conserved domain search. The GO terms for PVX_114890 of molecular function and cellular component were GO:0004252 (serine-type endopeptidase activity) and GO:0016021 (integral to membrane) respectively. Therefore SMAT80 correctly predicted it to be protease and we conclude that it is a putative rhomboid protease. A complete list of apicomplexan hypothetical proteins whose subject hits (against SwissProt using any of the three matrices) were probable or known proteases has been provided in Additional File 13. The GRAVY (grand average of hydropathy) values were calculated for these SMAT80-detected proteases (described in Methods). Out of the 17 proteases, four proteases; TP01_0999 (1.041), ETH_00005295 (0.301), TA05135 (0.244) and ETH_00042245 (0.049) had the positive GRAVY values indicating their hydrophobic nature while the remaining 13 probable proteases had negative values ranging from -0.013 (PVX_114890) to -1.421 (PY06720) indicating their hydrophilic nature (Additional File 14). The rhomboid proteases are integral to membrane and we expect them to have positive GRAVY values or very low negative GRAVY values. Six SMAT80 predicted proteases (TP01_0999, ETH_00005295, TA05135, ETH00042245, PVX_114890 and ETH_00006170) with positive or very low negative GRAVY values have stronger possibility of being rhomboid proteases compared to others (Additional File 14).

### Apicomplexan proteins features

In our previous study, we have shown that the amino acid compositions of proteins of nine apicomplexan species (*P. berghei, P. chabaudi, P. knowlesi, P. vivax, P. yoelii yoelii, T. gondii, C. parvum, T. parva *and *N. caninum*) were similar to that of *P. falciparum *proteins [10]. We carried out similar amino acid composition study [10] for all the 15 apicomplexan genomes and observed that all the apicomplexan genomes are having unusual amino acid composition like that of *P. falciparum *(data not shown) in comparison to *Mycobacterium tuberculosis *proteins. As discussed earlier, SMAT80 uniquely detected orthologs for 1374 apicomplexan hypothetical proteins and predicted 70 kinases and 17 proteases out of these hypothetical proteins. We compared the amino acid composition of these SMAT80 predicted kinases and proteases with that of yeast kinases and proteases respectively in terms of p-values (described in Methods). These apicomplexan proteins had very similar amino acid composition in terms of positively charged amino acids *i.e*. p-values were 0.88 and 0.90 for apicomplexan kinases and proteases respectively (Additional File 15). SMAT80-predicted apicomplexan kinases and proteases differed significantly from yeast kinases and proteases respectively in terms of composition of non-polar and negatively charged amino acids (Additional File 15) and we think that this is one of the reasons that BLOSUM matrices could not detect orthologs for these proteins.

## Conclusion

The available genomes of apicomplexan parasites have significant number of hypothetical proteins and improving the annotation of these proteins is one of the most important and challenging tasks of post-genomic era. We think one of the probable reasons for this was that the standard matrices (BLOSUM & PAM) could not detect orthologs for many compositionally-biased apicomplexan proteins [9,10]. We were able to find orthologs for 1374 such apicomplexan hypothetical proteins against SwissProt database using SMAT80 matrix in the BLAST searches. The subject annotations of these 1374 apicomplexan hypothetical proteins included 70 kinases, 14 phosphatases, 3 heat shock proteins, 17 proteases and several other important proteins therefore SMAT80 assigned some probable functions to these hypothetical proteins. The conserved domain search at NCBI site did not find any kinase domain in these 70 SMAT80-predicted kinases but found one rhomboid protease among the 17 SMAT80-predicted proteases. However further analysis of one of the predicted kinases (PY07003) revealed that the key functional residues of kinase domain were conserved in this protein. Similarly, one of the proteases (PVX_114890) was integral to membrane and having serine-type endopeptidase activity and these two features are the characteristics of the rhomboid proteases. Therefore SMAT80 correctly predicted it to be a protease and we conclude that it is a putative rhomboid protease. The hydrophobicity/hydrophilicity in terms of GRAVY values was also calculated for these SMAT80 predicted apicomplexan kinases and proteases. These probable apicomplexan kinases and proteases had significantly different non-polar and negatively charged amino acids contents in comparison to yeast kinases and proteases respectively and we think this was one of the reasons that BLOSUM matrices could not detect ortholog for these proteins. We also studied the performance of apicomplexan parasite-specific matrices in terms of ROC curves, an important aspect missing in our earlier studies [9,10]. These ROC curves indicated the higher specificity of SMAT80 matrix even against PSI-BLAST searches using compositionally adjusted BLOSUM62 matrix thereby signifying the role of these parasite-specific matrices in BLAST searches for apicomplexan proteins. And this higher specificity of SMAT80 matrix was studied in biological context also *i.e*. SMAT80 gave BLAST hits with very poor E-values and/or bit scores (compared to BLOSUM62) for the experimentally identified coccidia specific oocyst wall proteins against hematozoan parasites which are supposed not to have oocyst wall proteins. We have provided the lists of apicomplexan hypothetical proteins to which SMAT80 could assign some function in the supplementary material. We hope that this data would be useful for the researchers working on apicomplexan parasites in general and particularly for those working on apicomplexan kinases and proteases.

## Materials and methods

### Datasets used

PiroplasmaDB version 1.1 [26] data was used for *B. bovis, T. annulata *and *T. parva*, PlasmoDB release 8.0 [27,28] data for *P. berghei, P. chabaudi, P. falciparum, P. knowlesi, P. vivax *and *P. yoelii yoelii*, ToxoDB release 7.0 [29] data for *E. tenella, N. caninum *and *T. gondii*, CryptoDB release 4.3 [30] data for *Cryptosporidium hominis, C. muris *and *C. parvum*, the whole protein datasets from NCBI ftp site were used for rest other organisms used in this study and SwissProt/Uniprot database was downloaded from EBI ftp site.

### Software/programs used

The pairwise alignments using BLOSUM62 and PfFSmat60 matrices were carried out using ApicoAlign web server (http://www.cdfd.org.in/apicoalign/) developed by us. The blastp program of standalone BLAST software was used for carrying out local BLAST searches [31] (ftp://ftp.ncbi.nih.gov/toolbox/ncbi_tools/old/20051206). SMAT series of matrices were accepted by blastp program after some modifications in the source code [9,10]. The default gap open and extension penalties were used for BLOSUM62 while for BLOSUM90 and SMAT80, 10 and 1 were gap open and extension penalties respectively (best parameters for matrices which have entropies similar to BLOSUM90). Shell scripts were written using awk, sed and perl to find Best Bidirectional Hits between two organisms, best non-self hits common to two matrices and for other small purposes. The two tailed P-values for amino acid fractions (as correlated samples) were calculated using VassarStats (http://vassarstats.net/), a website for statistical computation. R package (version 2.10.1, http://www.r-project.org/) was used for various calculations and making graphs.

### Database searches

The BLAST searches (blastp program) were carried out for all the proteins of 15 apicomplexan parasites using SMAT80, BLOSUM90 and BLOSUM62 matrices against SwissProt database. These hits were classified into eight categories (1) better or similar E-values, better or similar scores and better or similar % identity with SMAT80 compared to BLOSUM90, (2) better or similar E-values, better or similar scores and poor % identity, (3) better or similar E-values, poor scores and better or similar % identity, (4) better or similar E-values, poor scores and poor % identity, (5) poor E-values, better or similar scores and better or similar % identity, (6) poor E-values, better or similar scores and poor % identity, (7) poor E-values, poor scores and better or similar % identity and (8) poor E-values, poor scores and poor % identity. Only the best non-self hits were considered for calculating the percentage of proteins for each category for all the 15 apicomplexan parasites.

### ROC curves

A unique dataset of all *P. berghei *and *P. yoelii *proteins with an assigned gene ontology was constructed and all *P. berghei *vs. all *P. yoelii *BLAST searches were carried out using BLOSUM62, BLOSUM90, SMAT80 and compositionally adjusted (BL62adj) matrices. The standalone PSI-BLAST searches were performed using blastpgp program of NCBI BLAST software with option -t 2 for compositionally adjusted BLOSUM62 matrix. The BLAST hits (e-value cut-off 1e-10) ranked by bit score were compared using GO identifiers for each pair of the query and subject sequences. Only those hits where the query and the subject proteins share gene ontologies were considered as true positives (TP) and the remaining hits were considered as false positives (FP). The numbers of false positives and true positives were used to make ROCn curves and for every curve we calculated the area under curve (AUCn). Here, n was chosen to be 162 as this was the maximum number of false positives which were present in all searches (BLOSUM62, BLOSUM90, SMAT80 and BL62adj).

### Calculation of hydropathy values

The average hydropathy values for SMAT80-detected apicomplexan protein kinases and proteases were calculated using "Sequence Manipulation Suite" (http://www.bioinformatics.org/sms2/protein_gravy.html). It gives "Protein GRAVY" (grand average of hydropathy) values for protein sequences. The GRAVY values are calculated by adding the hydropathy value for each amino acid and dividing it by the length of the sequence. The algorithm for calculating the values is based on the method developed by Kyte and Doolittle [32]. The grand average hydropathicity index for a protein indicates its solubility, with the positive GRAVY indicating hydrophobicity and negative GRAVY indicating hydrophilicity. The hydrophobicity profiles in Figure 5B were constructed using AlignMe tool [33] (http://www.bioinfo.mpg.de/AlignMe/index.html).

### Amino acid composition study of apicomplexan proteins

The amino acid compositions in terms of P-values for 15 apicomplexan parasites (used in this study) were calculated using the same methodology described earlier by us [10]. The amino acids were used as four categories: non-polar, polar with no charge, positively charged and negatively charged amino acids (see [10] for details). The protein sequences in FASTA format for yeast kinases and proteases were downloaded from AmiGO version 1.8 [34]. The amino acid composition of 70 SMAT80 predicted apicomplexan kinases was compared with that of yeast kinases and similarly for 17 SMAT80 predicted apicomplexan proteases it was compared with that of yeast proteases.

## Competing interests

The authors declare that they have no competing interests.

## Authors' contributions

JA carried out database searches against SwissProt, genome-wise BLAST searches, amino acid composition study, made ROC curves and wrote the manuscript. SRT calculated average hydropathy values, helped in compiling the results and writing the manuscript. AR co-ordinated and supervised the study. The final manuscript was read and approved by all the authors.

## Supplementary Material

Additional file 1 SMAT80 gives poor E-values for coccidian specific proteins in non-coccidian parasites. BLAST searches for coccidian-specific oocyst wall proteins of *Cryptosporidium parvum *were carried out against the hematozoans (non-coccidian) and coccidian apicomplexan parasites using BLOSUM62 and SMAT80 matrices. SMAT80 correctly gave poor E-values and/or bit scores for BLAST hits of these coccidian-specific proteins in hematozoans. Click here for file

Additional file 2 Genome-wise BLAST searches for apicomplexan proteins against 1215 bacterial species. The genome-wise BLAST searches were carried out for all the proteins of 15 apicomplexan species studied here against 1215 bacterial species using SMAT80, BLOSUM90 and BLOSUM62 matrices. Click here for file

Additional file 3 Number of hits found at different E-value thresholds for apicomplexan proteins in genome-wise BLAST searches against one another. The genome-wise BLAST searches were carried out for all the proteins of 15 apicomplexan species against one another using SMAT80, BLOSUM90 and BLOSUM62 matrices. Click here for file

Additional file 4 Comparison of performance of SMAT80 with that of BLOSUM90. We carried out BLAST searches for all the proteins of 15 apicomplexan parasites using SMAT80 and BLOSUM90 matrices against SwissProt database. An identical hit (best non-self) was assigned to one of the eight categories (1) better or similar E-values, better or similar scores and better or similar % identity with SMAT80 compared to BLOSUM90, (2) better or similar E-values, better or similar scores and poor % identity, (3) better or similar E-values, poor scores and better or similar % identity, (4) better or similar E-values, poor scores and poor % identity, (5) poor E-values, better or similar scores and better or similar % identity, (6) poor E-values, better or similar scores and poor % identity, (7) poor E-values, poor scores and better or similar % identity and (8) poor E-values, poor scores and poor % identity. As evident in the figure, most apicomplexan proteins fall in 1 & 7 categories that means SMAT80 performs better. Click here for file

Additional file 5 Apicomplexan proteins for which hits were detected against SwissProt database by SMAT80 but not by BLOSUM62 or BLOSUM90 matrices. This is the list of 1374 apicomplexan hypothetical proteins which did not give any BLAST hit against SwissProt database using BLOSUM series of matrices but SMAT80 was able to detect hits against SwissProt for these proteins. Click here for file

Additional file 6 List of 70 probable apicomplexan protein kinases detected by SMAT80 but not by BLOSUM series of matrices. This is the list of 70 apicomplexan hypothetical proteins whose SwissProt hits have probable or known kinase annotation. These hits were detected against SwissProt database by SMAT80 but not by BLOSUM series of matrices. Click here for file

Additional file 7 Results of batch Conserved Domain search for 70 predicted (by SMAT80) apicomplexan protein kinases. The protein sequences in FASTA format of these 70 apicomplexan hypothetical proteins were used for Conserved Domain search at NCBI site. Only 8 proteins gave hits and no kinase domain was detected. Click here for file

Additional file 8 Pair-wise alignments of probable apicomplexan protein kinases with a known *P. falciparum *protein kinase. The pairwise alignments were carried out using BLOSUM62 and PfFSmat60 matrices at ApicoAlign (http://www.cdfd.org.in/apicoalign) server. 30 SMAT80-predicted kinases (out of 70 of Supplementary Table 5) were used as query proteins and PF11_0220 as subject protein. *P. falciparum *protein kinase PF11_0220 is an experimentally known kinase (protein kinase activity GO:0004672, evidence code IDA, source: PlasmoDB version 9.0). Click here for file

Additional file 9 List of hypothetical apicomplexan proteins whose SwissProt hits are probable or known kinases. The BLAST hits obtained using SMAT80, BLOSUM90 & BLOSUM62 matrices against SwissProt database were pooled together into one set and the apicomplexan hypothetical proteins whose subject annotations include 'kinase' were filtered out of this set. We expect this list to be useful for the researchers working on apicomplexan kinomes. Click here for file

Additional file 10 The hydropathy values of 70 apicomplexan hypothetical proteins or SMAT80 predicted kinases. The GRAVY (grand average of hydropathy) values were calculated for 70 SMAT80 predicted apicomplexan kinases. Positive GRAVY indicates hydrophobicity and negative GRAY hydrophilicity. Click here for file

Additional file 11 List of 17 apicomplexan hypothetical proteins (or proteases as predicted by SMAT80) whose hits were detected by SMAT80 but not by BLOSUM series of matrices. This is the list of 17 apicomplexan hypothetical proteins whose SwissProt hits have probable or known protease annotation. These hits were missed by BLOSUM series of matrices but detected by SMAT80 matrix. Click here for file

Additional file 12 Results of batch Conserved Domain search for 17 predicted (by SMAT80) apicomplexan proteases. The Conserved Domain search in batch mode at NCBI site for these 17 apicomplexan proteins gave hits only for 5 proteins and rhomboid superfamily of proteases was detected. Click here for file

Additional file 13 List of hypothetical apicomplexan proteins whose SwissProt hits are probable or known proteases. The BLAST hits obtained using SMAT80, BLOSUM90 & BLOSUM62 matrices against SwissProt database were pooled together into one set and the apicomplexan hypothetical proteins whose subject annotations include 'protease' were filtered out of this set. We expect this list to be useful for the researchers working on role of proteases in apicomplexan biology. Click here for file

Additional file 14 The hydropathy values of 17 apicomplexan hypothetical proteins or SMAT80 predicted proteases. The GRAVY (grand average of hydropathy) values were calculated for 17 SMAT80 predicted apicomplexan proteases. Positive GRAVY indicates hydrophobicity and negative GRAY hydrophilicity. Click here for file

Additional file 15 Amino acid composition of SMAT80 predicted apicomplexan kinases and proteases compared to yeast kinases and proteases. The SMAT80 predicted apicomplexan kinases and proteases significantly differ from yeast kinases and proteases respectively in terms of non-polar and negatively charged amino acids content. We think this was one of the reasons that standard BLOSUM matrices could not detect orthologs for these proteins against SwissProt database. Click here for file
