# Supplementary figures and images for: The parasite specific substitution matrices improve the annotation of apicomplexan proteins
Source: BMC Genomics. 2012 Dec 7;13(Suppl 7):S19. doi: 10.1186/1471-2164-13-S7-S19 (PMC3521392; doi:10.1186/1471-2164-13-S7-S19)

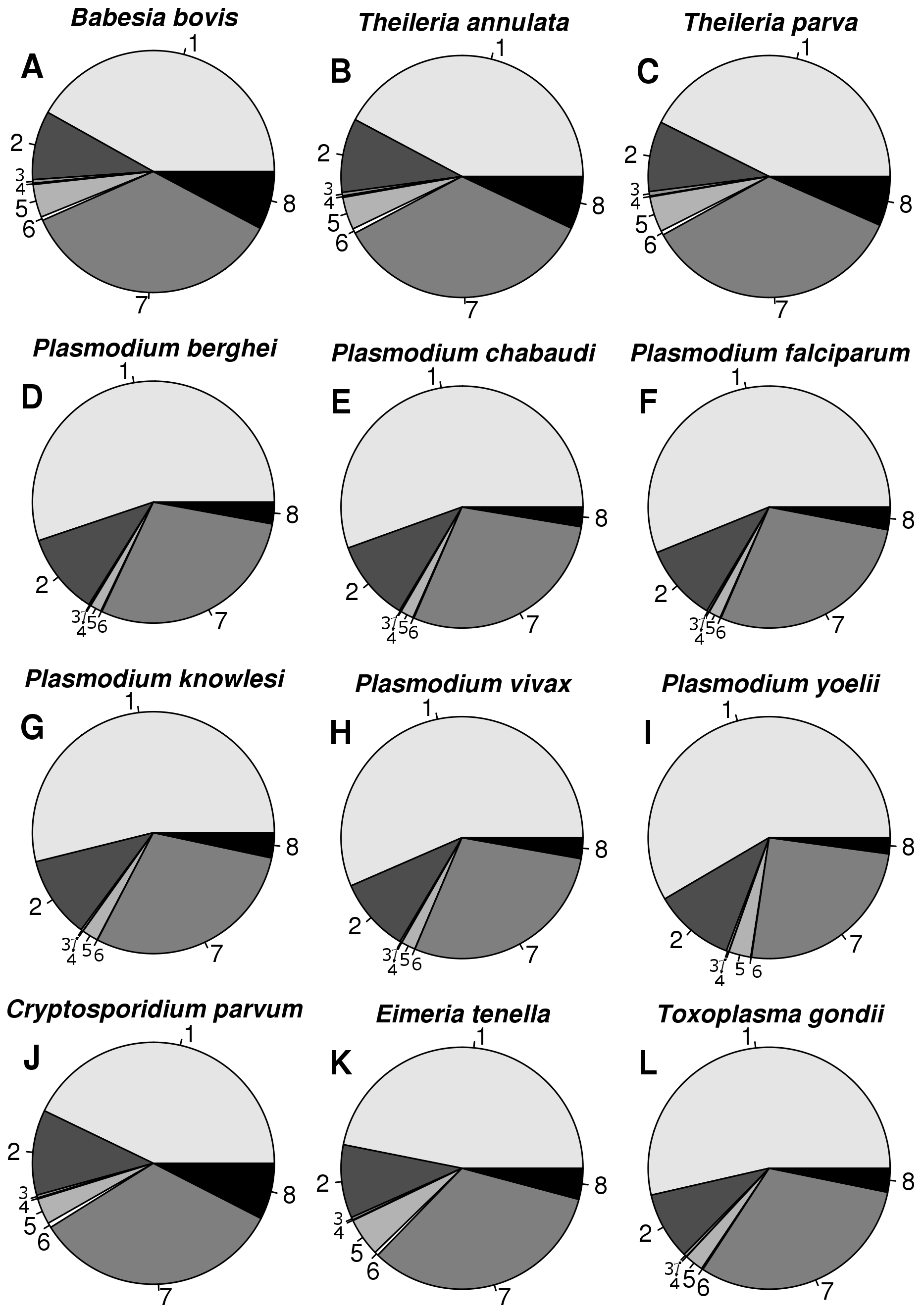

Supplement: Additional file 4 — Comparison of performance of SMAT80 with that of BLOSUM90. We carried out BLAST searches for all the proteins of 15 apicomplexan parasites using SMAT80 and BLOSUM90 matrices against SwissProt database. An identical hit (best non-self) was assigned to one of the eight categories (1) better or similar E-values, better or similar scores and better or similar % identity with SMAT80 compared to BLOSUM90, (2) better or similar E-values, better or similar scores and poor % identity, (3) better or similar E-values, poor scores and better or similar % identity, (4) better or similar E-values, poor scores and poor % identity, (5) poor E-values, better or similar scores and better or similar % identity, (6) poor E-values, better or similar scores and poor % identity, (7) poor E-values, poor scores and better or similar % identity and (8) poor E-values, poor scores and poor % identity. As evident in the figure, most apicomplexan proteins fall in 1 & 7 categories that means SMAT80 performs better. [file 1471-2164-13-S7-S19-S4.tiff]
