# Supplementary material for: The parasite specific substitution matrices improve the annotation of apicomplexan proteins
Source: BMC Genomics. 2012 Dec 7;13(Suppl 7):S19. doi: 10.1186/1471-2164-13-S7-S19 (PMC3521392; doi:10.1186/1471-2164-13-S7-S19)
Supplement: Additional file 8 — Pair-wise alignments of probable apicomplexan protein kinases with a known P. falciparum protein kinase. The pairwise alignments were carried out using BLOSUM62 and PfFSmat60 matrices at ApicoAlign (http://www.cdfd.org.in/apicoalign) server. 30 SMAT80-predicted kinases (out of 70 of Supplementary Table 5) were used as query proteins and PF11_0220 as subject protein. P. falciparum protein kinase PF11_0220 is an experimentally known kinase (protein kinase activity GO:0004672, evidence code IDA, source: PlasmoDB version 9.0). [file 1471-2164-13-S7-S19-S8.doc]

## Supplementary Table 7 - Pair-wise alignments of probable apicomplexan protein kinases with a known *P. falciparum* protein kinase.

## *P. falciparum* protein kinase PF11_0220 is an experimentally characterized kinase (Molecular Function GO:0004672, protein kinase activity, evidence code IDA, source: PlasmoDB version 9.0).

| **EMBOSS/water program** | **BLOSUM62 matrix** | | **PfFSmat60 matrix** | |
| --- | --- | --- | --- | --- |
| Protein | Length | Similarity (%) | Length | Similarity (%) |
| PY03697  PY07098  PY05947  PY04338   PY04864  PY04239  PY04679  TA10855   PY04991  TGME49_054830  TA20800   TA09360  TA11985  TA13235  TA13255  PY00070  PY05683  PY05393   TA17490  PVX_002695  TGME49_015660  PY00933  PKH_102260   PY04509   TA04215  PY04595  ETH_00034105   PVX_092835  PY03586  PY05197   PY07003 | 703  1290  285  947  1461  1584  625  1359  430  130  1736  777  808  777  777  1138  659  245  226  154  28  258  269  352   1395  1154  34  420  805  799  675 | 39.10  34.40  40.70  36.30  36.40  34.50  38.70  34.60  42.10  46.20  32.50  34.50  33.80  32.00  32.00  35.10  39.80  39.20  31.00  44.20  50.00  38.80  30.90  40.30  34.00  34.90  55.90  33.30  36.30  36.30  36.20 | 1094  1649  449  1577  1746  1704  697  1772  659  1846  1965  1106  1525  1477  1477  1567  1182  438  467  886  838  427  565  672  1921  1714  1342  726  1235  1458  875 | 43.10  47.10  44.80  36.80  45.30  48.20  47.90  44.60  49.90  42.00  49.10  39.40  39.90  35.70  35.70  45.40  39.00  40.40  37.5  38.10  35.80  38.60  31.90  37.80  45.80  44.80  29.30  38.20  46.30  38.70  40.80 |
